# Evaluation of *Salvia yangii* Extract as a Promising Protective Raw Material Applied Topically to the Skin

**DOI:** 10.3390/molecules30173505

**Published:** 2025-08-27

**Authors:** Monika Michalak, Martyna Zagórska-Dziok, Paulina Żarnowiec, Aneta Ostróżka-Cieślik, Anita Bocho-Janiszewska, Małgorzata Stryjecka, Natalia Dobros, Dorota Kostrzewa, Katarzyna Paradowska

**Affiliations:** 1 Department of Pharmaceutical Sciences, Collegium Medicum, Jan Kochanowski University, IX Wieków Kielc 19, 35-317 Kielce, Poland; 2 Department of Technology of Cosmetic and Pharmaceutical Products, Medical College, University of Information Technology and Management in Rzeszów, Sucharskiego 2, 35-225 Rzeszów, Poland; mzagorska@wsiz.edu.pl; 3 Department of Microbiology, Faculty of Natural Sciences, Jan Kochanowski University, Uniwersytecka 7, 25-406 Kielce, Poland; paulina.zarnowiec@ujk.edu.pl; 4 Department of Pharmaceutical Technology, Faculty of Pharmaceutical Sciences in Sosnowiec, Medical University of Silesia, 41-200 Sosnowiec, Poland; aostrozka@sum.edu.pl; 5 Faculty of Applied Chemistry, Casimir Pulaski Radom University, ul. Chrobrego 27, 26-600 Radom, Poland; a.janiszewska@uthrad.pl; 6 Department of Dietetics, The University College of Applied Sciences in Chełm, Pocztowa 54, 22-100 Chełm, Poland; mstryjecka@panschelm.edu.pl; 7 Department of Organic and Physical Chemistry, Faculty of Pharmacy, Medical University of Warsaw, Banacha 1, 02-097 Warsaw, Poland; katarzyna.paradowska@wum.edu.pl; 8 Łukasiewicz Research Network—New Chemical Syntheses Institute, Al. Tysiąclecia Państwa Polskiego 13A, 24-110 Puławy, Poland; dorota.kostrzewa@ins.lukasiewicz.gov.pl

**Keywords:** *Salvia yangii*, bioactive compounds, antioxidant properties, antimicrobial activity, photoprotective effect, anti-collagenase, anti-elastase, anti-tyrosinase, hydrogel, lotion

## Abstract

*Salvia yangii* is a popular garden plant known for its medicinal properties. The aim of this study was to evaluate the chemical composition and skin protective properties of *S. yangii* extracts, which have not previously been studied in this regard. Comparison of the water–ethanol extract obtained by ultrasound-assisted extraction (UAE) with a CO_2_ extract showed that the former had higher content of polyphenolic compounds. Chromatographic analyses of UAE identified such phenolic compounds as rosmarinic acid, hesperidin, and caffeic acid. The biological properties of UAE were also tested in vitro on 15 microbial strains as well as on two lines of skin cells. In addition, a hydrogel and lotion based on the extract were tested for rheological and textural properties. This study showed that *S. yangii* extract can be a valuable natural cosmetic material owing to its antimicrobial, antioxidant, photoprotective, and anti-aging effects. The future use of *S. yangii* extracts in the cosmetic industry is promising due to its particular chemical profile and biological properties.

## 1. Introduction

In recent years, there has been an increasing interest in cosmetics based on natural raw materials with skincare properties. With the growing trend that plant-based ingredients can support or even replace synthetic chemicals in cosmetic products, they are becoming increasingly important as ingredients in skin care products. Among the advantages of natural cosmetics mentioned by consumers are their gentle action on the skin, effectiveness, non-toxicity, lack of side effects with long-term use, and the fact that they are environmentally friendly. Equally important is the multifaceted, positive effect of plants on the skin, resulting from the content of various biologically active compounds. Plants are widely used as antioxidant, anti-inflammatory, antibacterial, photoprotective, brightening, and anti-aging agents. Plant extracts used as cosmetic ingredients can be obtained from the whole plant or its parts (fruits, leaves, roots, bark, stems, branches, seeds, or flowers) by extraction using a properly selected solvent, e.g., water, ethyl alcohol, glycerin, or glycols [1,2]. The cosmetics industry is paying increasing attention to “green” solvents and extraction techniques for extracting phytoconstituents from natural sources. Ultrasound-assisted extraction (UAE) and supercritical and subcritical fluid extraction (SFE) can be innovative alternatives to conventional extraction processes (i.e., Soxhlet extraction, maceration, and water distillation) [3].

**Figure 1:**
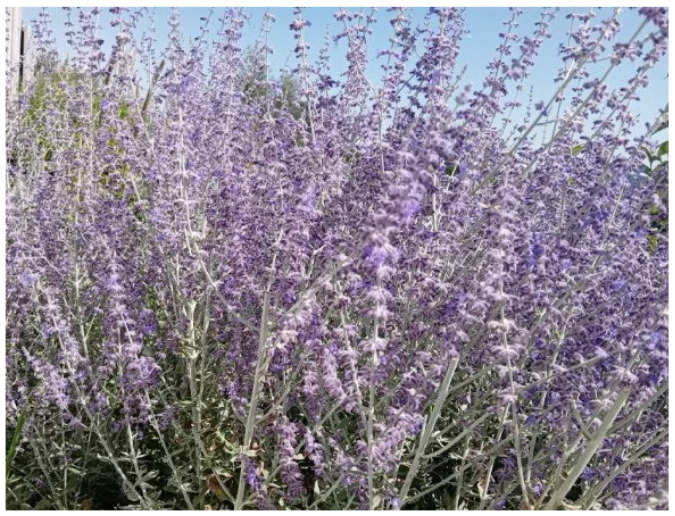
*Salvia yangii* B.T. Drew (photo by M. Michalak).

*Salvia yangii* B.T. Drew (formerly *Perovskia atriplicifolia* Benth.) is an ornamental plant with a strong, pleasant scent from the subgenus *Perovskia* and family Lamiaceae. The species, popularly known as Russian Sage, is native to southwestern and central Asia and cultivated worldwide. It is a perennial, shrubby herb that grows to a height of 1.0–1.5 m and flowers from July into late October. It has square stems, grayish-green leaves, and blue to purple flowers covered with silvery hairs (Figure 1) [4,5,6,7].

*S. yangii* has been used in traditional medicine to prevent and cure various diseases. However, little is known of the medicinal value of the plant. Most published data concern the composition and activity of essential oil from the aerial parts. There are only a few scientific studies in the literature confirming the biological effects of plant extracts, such as anti-inflammatory [4], antidiabetic [8], or anti-cholinesterase activity [5,6]. Polyphenolic compounds (e.g., perovskoate, perovskoside, caffeic, and ferulic acid), as well as diterpenoids (przewalskin E, 1α-hydroxypisiferanol, brussonol, and perovskatones A, B, C, and D) and triterpenoids (atricins A and B, 2α,3β,24-trihydroxyolean-12-en-28-oic acid and 2α,3β,19β-trihydroxyurs-12-en-28-oic acid), are responsible for the biological properties [4,5,7,9,10]. Promising results on the potential external use of another species, *P. abrotanoides*, were presented by researchers in Iran. The authors showed that ointment with this plant can accelerate wound healing by producing VEGF [11]. The ethnopharmacological importance of the genus *Salvia* and the scarcity of studies on the phytochemical composition and biological properties of *S. yangii* extract prompted us to conduct further research on this species.

*S. yangii* is a little-known and insufficiently researched plant. Only limited studies on the phytochemical composition and biological activity of *S. yangi* extracts can be found in the literature. To the best of our knowledge, there is no available data on the content of the main groups of bioactive compounds in the extracts obtained by ultrasound-assisted extraction (UAE) and supercritical fluid extraction (SFE). In this study, in addition to the comparative assessment of two extracts from *S. yangii* herb obtained using solvents significantly different in polarity, an extended phytochemical analysis using HPLC methods and assessment of skin-relevant properties were carried out for UAE. Moreover, the effect of the water–ethanol extract on cell viability, UVB protection, and antioxidant and anti-aging properties was assessed in two skin cell lines. The antimicrobial properties and antibiofilm activity were assessed against 14 bacterial and 1 fungal strain. In addition, an important aspect of this article in the context of the possible use of *S. yangii* extract in cosmetics is the rheological and textural characterization of the self-developed and self-prepared model formulations.

## 2. Results and Discussion

### 2.1. Phytochemical Constituents of Extracts

The first step of this study was to conduct a comparative chemical analysis of two extracts from the *S. yangii* aerial parts, obtained using solvents that differ significantly in polarity. Spectrophotometric methods were used to assess the content of bioactive compounds in a water–ethanol *S. yangii* extract obtained by ultrasound-assisted extraction (UAE), which was compared to an extract obtained by supercritical fluid extraction (SFE) (Table 1). Due to their mechanism of action, both methods are classified as “green” chemistry. The former method employs ultrasonic waves to enhance the process, thereby increasing the extraction efficiency by accelerating the transfer of substances to the solvent. The second method involves the extraction of substances from raw materials using a supercritical fluid (most commonly carbon dioxide). The selectivity of CO_2_ in its supercritical state facilitates the production of pure extracts free of harmful solvents, allowing for high yields while minimizing the risk of thermal sample transformation [3]. This is of particular significance when the extract is to be used as a cosmetic ingredient.

Literature data indicate that most studies relating to the chemical composition concern the essential oil from *S. yangii* [7], while the analysis of phytochemical profiles of non-volatile compounds is rather limited. The research conducted so far has focused on the evaluation of extracts from roots [12,13], leaves [9,12], or the aerial parts [4,5] of the plant. A review of the available data also indicates that researchers used different methods and solvents for extraction [4,5,8,9,12,13]. Due to the lack of studies evaluating water–ethanol extracts obtained by ultrasound-assisted extraction (UAE) and supercritical fluid extraction (SFE), we conducted such analyses to fill this gap. In terms of practical use of the tested *S. yangii* extracts in natural cosmetics, solvents and extraction methods that align with the idea of green chemistry were selected. Water and supercritical carbon dioxide (CO_2_) are “green” solvents that are essential for the extraction of phytoconstituents from natural sources. Both water and supercritical CO_2_ have no harmful effects on health or the environment. The polarity of water allows it to be used as an extraction solvent for both natural and inorganic water-soluble substances. A disadvantage of this solvent is the lower solubility of non-polar molecules in water, although this problem can be partially resolved by using a mixture of alcohol and water. Alcohols such as methanol, ethanol, and isopropyl alcohol have similar solvent properties, but due to its non-toxicity, ethanol is recommended for use in cosmetic products [3]. The results of this study confirm that ultrasound-assisted extraction is better suited for the extraction of polar compounds, while supercritical carbon dioxide extraction is useful for the extraction of non-polar compounds. Research results revealed that the total content of polyphenolic, flavonoid, phenolic acids, and condensed tannins in UAE is higher than in SFE (Table 1). Based on the results of spectrophotometric analyses, the water–ethanol extract from *S. yangii* herb obtained by ultrasound-assisted extraction was used for further analysis.

**Figure 2:**
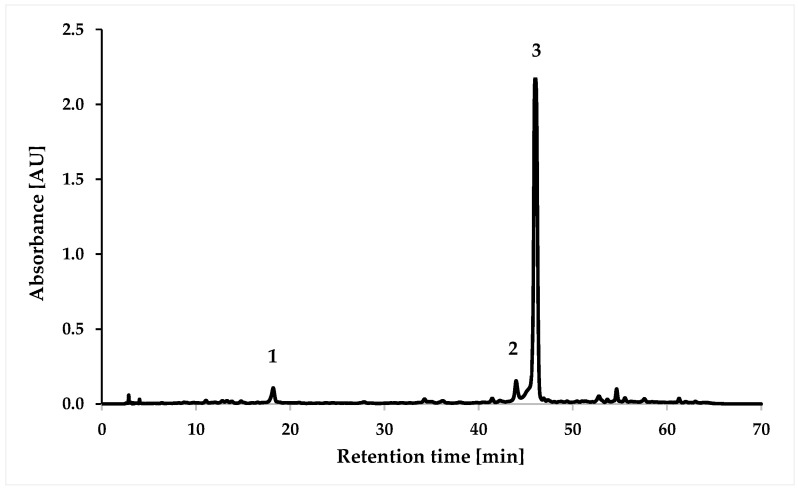
HPLC chromatogram at 310 nm for *S. yangii* water–ethanol extract (UAE). Numbering of polyphenolic compounds: 1—caffeic acid, 2—hesperidin, and 3—rosmarinic acid.

The HPLC chromatogram at 310 nm for the water–ethanol extract of *S. yangii* obtained by ultrasound-assisted extraction is presented in Figure 2. The identity of compounds was performed by comparing the retention times of the peaks and their UV-Vis spectra with corresponding standards (Figures S1 and S2).

The identified compounds were caffeic acid (no. 1), hesperidin (no. 2), and rosmarinic acid (no. 3). Other authors also confirmed the presence of rosmarinic acid [13,14], caffeic acid [15], and hesperidin [12]. The water–ethanol extract of *S. yangii* was characterized by the highest content of rosmarinic acid (1.32 mg/mL) and lower contents of hesperidin (0.16 mg/mL) and caffeic acid (0.04 mg/mL) (Table 2). The obtained values of the coefficient of determination (R^2^) higher than 0.998 indicate high linearity, while the LOD values in the range from 0.01 to 0.08 and the LOQ values from 0.02 to 0.23 confirm the usefulness of the applied method for the quantification of compounds (Table S1).

### 2.2. Antioxidant Activity of Extract

In the present study, antioxidant activity was determined by two spectrophotometric methods, i.e., the DPPH (1,1-diphenyl-2-picrylhydrazyl) and FRAP (ferric-reducing antioxidant potential). The ability to scavenge DPPH radicals was 71.89 ± 0.24% (percent inhibition of DPPH radical), while the antioxidant properties determined by the FRAP method were at the level of 1.64 ± 0.01 mmol/L. However, the obtained values were significantly lower than those obtained for the positive control—rosmarinic acid (96.83 ± 0.04% for DPPH assay and 9.25 ± 0.01 mmol/L for FRAP assay). To the best of our knowledge, there are no studies evaluating the antioxidant properties of water–ethanol extracts of *Perovskia atriplicifolia* herb. However, Ghaffari et al. [16] investigated the antioxidant potential of methanolic leaf extracts of 17 populations of *Perovskia abrotanoides* collected from different geographical regions in Iran. Due to the high variability of the assessed populations, the antioxidant activity for 80% methanol extracts ranged from 34% to 73% [16]. However, the antioxidant activity obtained for our extract (71.89%) was within the range obtained for different populations of *P. abrotanoides* from Iran.

Many scientific reports have confirmed the antioxidant properties of rosmarinic acid [17,18], caffeic acid [19], and hesperidin [20]. Rosmarinic acid exhibits significant DPPH and ABTS radical scavenging activity. Zuo et al. [21] demonstrated that the IC50 value for rosmarinic acid (28.5 μmol/L in the DPPH assay and 6.82 μmol/L in the ABTS assay) was slightly lower than the IC50 value of dihydromyrcetin (12.4 μmol/L in the DPPH assay and 3.41 μmol/L in the ABTS assay), which exhibits strong antioxidant activity. The antioxidant activity of caffeic acid, which inhibited the DPPH radical in 84.63% at a 1 mM concentration, was similar to that of ascorbic acid (83.43% DPPH inhibition) [22]. In contrast, the antioxidant activity of hesperidin is slightly weaker. In the Al-Ashaal and El-Sheltawy study [23], hesperidin neutralized the DPPH radical by 36%, compared to 100% obtained for ascorbic acid. The antioxidant properties of polyphenolic compounds (rosmarinic acid, caffeic acid, and hesperidin) are related to their chemical structure, particularly the presence of an aromatic ring and the number and position of hydroxyl groups. Compounds containing more hydroxyl groups exhibit greater antioxidant activity. The antioxidant activity of polyphenols results from their ability to inactivate radicals by donating a hydrogen atom or an electron (FRAP) or by both mechanisms simultaneously (DPPH). Furthermore, polyphenols chelate transition metal ions, prevent oxidation by inducing antioxidant enzymes (peroxide dismutase, catalase, and glutathione peroxidase), and inhibit the activity of enzymes involved in the production of ROS (e.g., xanthine oxidase) [1,24,25].

### 2.3. Effect of Extract on Skin Cell Viability

**Figure 3:**
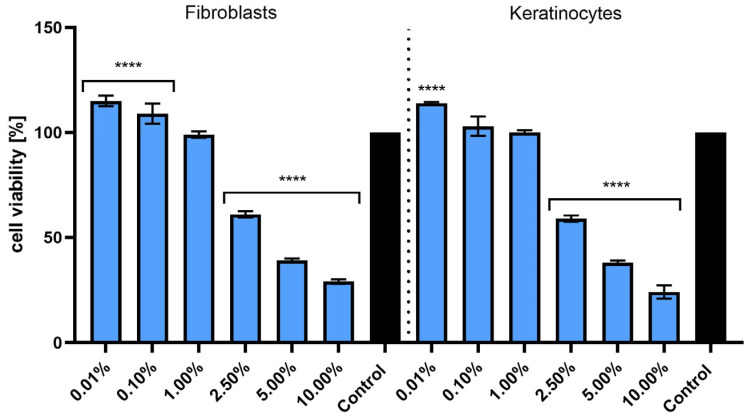
Effect of various concentrations of *S. yangii* water–ethanol extract (0.01–10.00%) on the viability of human dermal fibroblasts (HDFs) and keratinocytes (HaCaTs), after 24 h exposure, assessed using the Alamar Blue assay. The results are expressed as a percentage of viable cells relative to the untreated control (set as 100%). Values represent the mean ± SD from three independent experiments performed in triplicate. **** *p* < 0.0001 versus the control.

The first test used in this study (Alamar Blue assay) is a widely accepted method for evaluating cell viability and metabolic activity under in vitro conditions. This approach relies on the ability of living cells to reduce the non-fluorescent resazurin dye to the fluorescent and pink-colored compound resorufin as a result of mitochondrial and cytoplasmic enzymatic activity. The extent of this redox reaction serves as an indirect indicator of the number of metabolically active cells. Due to its high sensitivity, low cytotoxicity, and compatibility with both colorimetric and fluorometric detection, the Alamar Blue assay is frequently used to monitor cellular responses to bioactive substances, including natural extracts [26]. In both cell lines, low concentrations of the extract (0.01%, 0.10%, and 1.00%) did not negatively affect cell viability. A statistically significant increase in metabolic activity was observed at 0.01% in comparison to the untreated control (**** *p* < 0.0001), suggesting a potential stimulatory or hormetic effect at very low doses. Conversely, exposure to higher concentrations (2.50%, 5.00%, and 10.00%) resulted in a pronounced, concentration-dependent decrease in cell viability in both fibroblasts and keratinocytes. At 10.00%, viability dropped below 50% relative to the control in both cell types, indicating significant cytotoxicity (Figure 3). Statistical analysis confirmed that the reduction in viability at concentrations ≥ 2.50% was highly significant (**** *p* < 0.0001). Overall, these results indicate that *S. yangii* extract exerts a biphasic effect on skin cells: non-toxic or even stimulatory at low concentrations, but cytotoxic at higher doses. Such a profile is consistent with other plant-derived bioactive compounds that exhibit dose-dependent dual biological activity [27].

**Figure 4:**
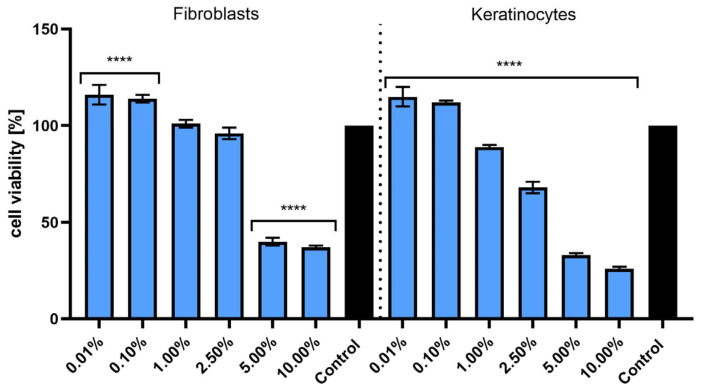
Effect of various concentrations of *S. yangii* water–ethanol extract (0.01–10.00%) on the viability of human dermal fibroblasts (HDFs) and keratinocytes (HaCaTs) after 24 h exposure, assessed using the Neutral Red assay. The results are expressed as a percentage of viable cells relative to the untreated control (set as 100%). Values represent the mean ± SD from three independent experiments performed in triplicate. **** *p* < 0.0001 versus the control.

The second test used in this study (Neutral Red) is a commonly used test to assess cell viability that involves the use of Neutral Red dye, which penetrates living cells and accumulates in their lysosomes [28]. For HDF fibroblasts, the highest cell viability was observed at concentrations of 0.01% and 0.10%. Increasing the concentration to 1.00% and 2.50% did not significantly affect cell viability compared to the control. However, at higher concentrations (5.00% and 10.00%), a significant decrease in cell viability was observed, with values dropping below 50% at 10.00%, indicating toxicity at higher concentrations. Similar to fibroblasts, keratinocytes exhibited the highest cell viability at 0.01% and 0.10% concentrations, with values close to the control. As the concentration of the extract increased, cell viability began to decrease, reaching the lowest level at 10.00%, where viability was less than 30% (Figure 4). According to the Neutral Red assay results, *S. yangii* extract shows a stimulatory effect at low concentrations (0.01% and 0.10%) for both HDF fibroblasts and HaCaT keratinocytes. However, at higher concentrations (5.00% and 10.00%), toxicity was observed, leading to a decrease in cell viability. These results suggest that using the extract at appropriate low concentrations could be beneficial for skin cell viability, while higher concentrations may cause cellular damage.

Our in vitro investigation revealed that *S. yangii* extract exhibits a biphasic, concentration-dependent effect on skin cell viability. At concentrations up to approximately 1%, no cytotoxicity was detected via Alamar Blue and Neutral Red assays, whereas higher concentrations induced a significant decrease in cell viability. This phenomenon aligns with the hormetic behavior often observed in plant-derived bioactive mixtures, where low doses may exert protective or neutral effects, while higher doses provoke cellular stress or toxicity [27,29].

*Salvia yangii* is known to harbor a diverse array of bioactive constituents—such as terpenoids, diterpenoids, triterpenoids, phenolic compounds, and essential oils—many of which contribute antioxidant, anti-inflammatory, neuroprotective, and other beneficial properties [7,30,31]. In lower concentrations, phenolic and terpenoid compounds may mitigate oxidative stress by scavenging reactive oxygen species or reinforcing mitochondrial function, thereby preserving cell metabolism [32,33]. At elevated doses, the same compounds might disrupt membrane integrity or shift into prooxidant activity, ultimately impairing mitochondrial processes and diminishing viability in a pattern consistent with redox-mediated cytotoxicity [34,35,36].

Comparable concentration-dependent cytotoxic effects have been documented in other *Salvia* species. For instance, *Salvia candidissima* Vahl. subsp. *candidissima* extract has been shown to exert anticancer effects against the MCF-7 and MDA-MB-231 breast cancer cell lines, with cytotoxicity levels dependent on the concentration used [37]. Moreover, decoctions of various *Salvia* species demonstrated potent cytotoxicity against carcinoma lines such as HepG2, HeLa, and MCF-7, an effect often attributed to pronounced levels of phenolic constituents and their modulatory impact on oxidative stress pathways [38].

In summary, the observed concentration-dependent effects may be explained by the dual action of the extract’s bioactive constituents. At low concentrations, the antioxidant components may enhance cellular resilience by mitigating oxidative stress and supporting mitochondrial function. In contrast, at higher concentrations, these constituents can compromise membrane integrity, promote the generation of reactive oxygen species, impair mitochondrial NAD(P)H-dependent reduction (as detected by the Alamar Blue assay), and decrease lysosomal uptake (as assessed by the Neutral Red assay), ultimately leading to reduced cell viability. Our findings indicate that low concentrations of the *S. yangii* extract exert a beneficial effect on skin cell viability, which is of particular relevance for its potential incorporation into topical formulations. Such concentrations may support skin cell health by mitigating oxidative stress and preserving mitochondrial function, thereby enhancing the safety profile of cosmetic or dermatological products intended for prolonged or repeated application.

### 2.4. Anti-Aging Activity of Extract

Research into the ability of plant extracts to inhibit enzymes like collagenase, elastase, and tyrosinase holds significant potential for applications in both cosmetology and dermatology. Collagenase and elastase are enzymes that break down collagen and elastin, which are key components for maintaining skin firmness and elasticity [39]. Tyrosinase, involved in melanin production, is crucial for pigmentation processes; its overactivity can lead to issues such as skin discoloration [40]. By understanding the inhibitory effects of plant extracts on these enzymes, it may be possible to develop skincare products that prevent the breakdown of skin structures and address pigmentation disorders.

**Figure 5:**
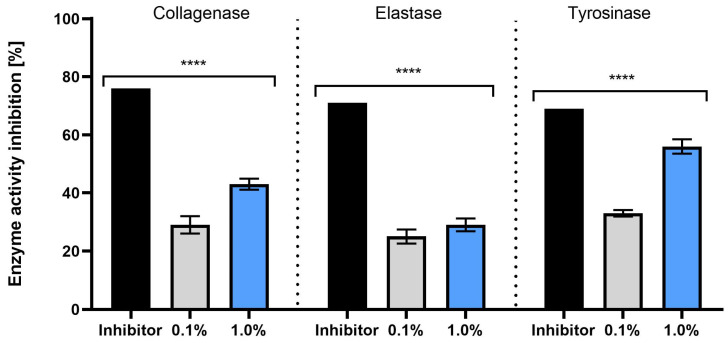
Inhibitory effects of the *S. yangii* water–ethanol extract at concentrations of 0.1% and 1.0% on collagenase, elastase, and tyrosinase activities, compared to positive control inhibitors (SPCK (30 µM) for elastase; 1,10-phenanthroline (1 mM) for collagenase; kojic acid (500 μg/mL) for tyrosinase). Results are expressed as percentage inhibition of enzyme activity. Data represent mean ± SD (*n* = 3). Statistically significant differences compared to the positive control are indicated (**** *p* < 0.0001).

Figure 5 shows how plant extracts influence the activity of collagenase, elastase, and tyrosinase at two different inhibitor concentrations (0.1% and 1.0%). Both concentrations show an inhibitory effect on these enzymes, with notable differences in efficacy. At the 1.0% concentration, a more significant reduction in collagenase activity is observed compared to the 0.1% concentration. The difference is statistically meaningful, suggesting that higher concentrations of the inhibitor are more effective in reducing collagenase activity. This effect may be valuable in protecting the skin against collagen degradation, a factor in the aging process. A similar trend is seen with elastase inhibition, where the 1.0% concentration leads to a more pronounced reduction in enzyme activity compared to the 0.1% concentration. The greater effect observed at the higher concentration could help preserve skin elasticity, making it relevant for formulations aimed at maintaining youthful skin appearance. Tyrosinase activity is also more effectively inhibited at the 1.0% concentration, showing that higher concentrations of the extract can significantly reduce tyrosinase’s action. In summary, the data highlights that increasing the concentration of the *S. yangii* extract leads to a stronger inhibition of these enzymes. This suggests that plant extracts with higher concentrations could be useful in the development of skincare treatments targeting the prevention of skin degradation and the management of pigmentation problems, offering valuable applications in both cosmetology and dermatology.

Based on the available scientific literature, there is limited but promising information regarding the inhibitory effects of *S. yangii* on key skin-related enzymes, including collagenase, elastase, and tyrosinase. While research on this specific plant remains relatively sparse, some studies have highlighted its potential in modulating enzyme activity and contributing to skin health. A study by Miroliaei et al. [8] investigated the methanolic extract of *P. atriplicifolia* roots and found that it exhibited inhibitory effects on both collagenase and elastase. This suggests that the plant may have a role in preventing the degradation of collagen and elastin, which are vital for maintaining the structural integrity and elasticity of the skin. To date, there have been no reports regarding the potential inhibition of tyrosinase activity by the studied extracts, and this study represents the first evidence of such an effect. The ability to inhibit melanin synthesis by *S. yangii* extract may suggest its potential application in formulations designed to protect against disorders related to excessive pigmentation and the formation of skin hyperpigmentation. The chemical composition of *P. atriplicifolia* is rich in various bioactive compounds, such as rosmarinic acid, diterpenoids, and phenolic acids, all of which are known for their broad biological activity [41,42,43,44]. Rosmarinic acid has been shown to contribute to the preservation of the extracellular matrix through the modulation of elastase and collagenase activity. In vitro experiments demonstrated a dose-dependent inhibition of porcine pancreatic elastase, with approximately 55% reduction at 60 µg/mL, an effect further supported by docking studies indicating interactions within the catalytic site of the enzyme [45]. In fibroblast models, rosmarinic acid and rosmarinic acid–rich extracts promoted type I collagen synthesis while attenuating the activity of matrix metalloproteinases such as MMP-1, MMP-2, and MMP-9 [46]. Moreover, studies in keratinocytes and fibroblasts exposed to oxidative stressors highlighted its ability to counteract enzyme activation associated with extracellular matrix degradation, further underscoring its protective role in skin homeostasis [47]. Collectively, these findings indicate that rosmarinic acid may act both as a direct elastase inhibitor and as a regulator of collagen turnover, supporting its relevance as a natural agent in maintaining dermal integrity. Importantly, rosmarinic acid and its methyl ester have also been reported to suppress mushroom tyrosinase activity in a competitive manner, with IC_50_ values in the micromolar range that are comparable to kojic acid, a standard reference inhibitor, further extending the potential applications of this compound in skin-related formulations [48]. The inhibitory activity of *S. yangii* extract against elastase, collagenase, and tyrosinase may also be related to the presence of terpenoids. A recent comprehensive review emphasized that numerous terpenoids, particularly diterpenoids, exhibit inhibitory activity against human neutrophil elastase, thereby highlighting their relevance as natural anti-inflammatory and tissue-protective agents [49]. In line with these observations, Andrade et al. [50] demonstrated that extracts and isolated constituents of *Plectranthus* species, many of which are terpenoid in nature, exert significant inhibitory effects on elastase, collagenase, and tyrosinase while also displaying antioxidant properties, suggesting a multi-targeted mode of action in skin protection. A broader review further highlights that diterpenoids and other terpenoid subclasses may act not only as direct inhibitors of these enzymes but also as antioxidants, offering a dual protective effect on extracellular matrix stability and pigmentation control [50]. Taken together, these findings suggest that diterpenoids contribute substantially to the enzyme-inhibitory potential observed for *S. yangii* extracts, reinforcing their relevance as bioactive constituents with anti-aging and skin-protective properties. Phenolic acids identified in *S. yangii*, such as caffeic, chlorogenic, protocatechuic, and p-hydroxybenzoic acids, also show promising effects on matrix-degrading enzymes and melanogenic activity [31]. Caffeic acid notably counteracted UVB-induced upregulation of MMP-1 in human dermal fibroblasts by suppressing both MMP-1 expression and downstream MAPK/NF-κB signaling, thereby reducing collagen degradation under photoaging conditions [51]. Protocatechuic acid demonstrated both in vitro and ex vivo efficacy in skin: it reduced MMP-1 secretion in UVA-irradiated dermal fibroblasts while stimulating type I collagen production, and topical formulations containing protocatechuic acid reduced wrinkle appearance in humans [52]. As for chlorogenic acid and p-hydroxybenzoic acid, while robust data on elastase or collagenase are limited, chlorogenic acid has been reported to suppress melanogenesis and modulate MMP expression in skin models, aligning with its role in oxidative stress relief and enzymatic regulation; p-hydroxybenzoic acid acts as a reversible competitive inhibitor of tyrosinase, though its potency is relatively modest (IC_50_ in the high-micromolar to millimolar range) [53,54]. Taken together, these phenolic acids appear to exert dual effects—indirect modulation of protease expression in skin cells and, to a lesser extent, direct enzyme inhibition—making caffeic and protocatechuic acids particularly notable candidates for anti-photoaging interventions. These compounds likely contribute to the observed inhibitory effects on enzymes, offering a potential mechanism through which the plant exerts its beneficial effects on skin health and appearance. In conclusion, while preliminary findings suggest that *S. yangii* has the potential to inhibit collagenase, elastase, and tyrosinase, further research is required to confirm these effects and fully understand the underlying mechanisms. If proven effective, this plant could become an important ingredient in dermatological and cosmetic formulations aimed at combating skin aging, degradation, and pigmentation issues.

### 2.5. Assessment of Photoprotective Activity of Extract on Keratinocytes and Fibroblasts

Excessive generation of reactive oxygen species (ROS) in skin cells caused by UVB radiation leads to oxidative stress, resulting in DNA damage, protein degradation, lipid peroxidation, and ultimately contributing to photoaging and skin carcinogenesis [55,56]. Therefore, protecting keratinocytes and fibroblasts—the main cellular components of the epidermis and dermis—from UVB-induced oxidative stress is crucial for maintaining skin integrity and preventing various skin disorders. In recent years, there has been growing interest in natural plant-derived compounds as effective photoprotective agents due to their ability to neutralize ROS and mitigate UV-induced cellular damage [56].

**Figure 6:**
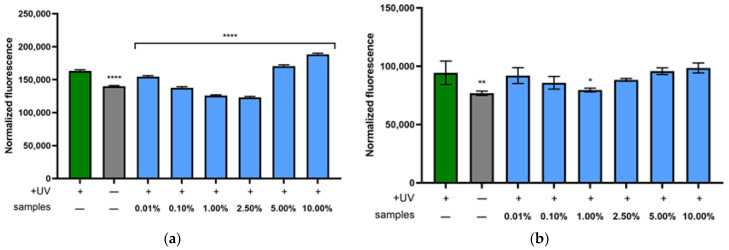
Intracellular reactive oxygen species levels in fibroblasts (**a**) and keratinocytes (**b**) following UVB (0.5 J/cm^2^) exposure and treatment with various concentrations (0.01–10.00%) of water–ethanol *S. yangii* extract. A 10 µM solution of the fluorescent probe H_2_DCFDA was used in the analysis. Green bars represent ROS levels in cells irradiated with UVB alone, gray bars indicate untreated control cells, and blue bars correspond to cells treated with different extract concentrations. Data are presented as mean ± SD (*n* = 3). **** *p* < 0.0001, ** *p* = 0.0031, and * *p* < 0.0446 versus the control.

Figure 6a illustrates the intracellular ROS levels in fibroblasts exposed to UVB radiation (green bar), which shows a substantial increase in oxidative stress compared to untreated control cells without UVB exposure (gray bar). Treatment with the water–ethanol extract of *S. yangii* at concentrations ranging from 0.01% to 2.50% significantly reduced ROS levels compared to the UVB-exposed group, with the most pronounced protective effects observed at 1.00% and 2.50%, where ROS levels were lower than the negative control. However, at higher concentrations of 5.00% and 10.00%, a notable increase in intracellular ROS was observed, suggesting increased oxidative stress. These findings indicate that the extract exhibits photoprotective, ROS-scavenging activity at lower concentrations, effectively shielding fibroblasts from UVB-induced oxidative damage, whereas concentrations exceeding 5.00% may induce oxidative stress and contribute to cytotoxic effects.

In the case of keratinocytes (Figure 6b), a weaker photoprotective effect was observed compared to the response seen in fibroblasts. The results demonstrated a statistically significant reduction in intracellular ROS levels only following treatment with the extract at a concentration of 1.00%. Lower concentrations resulted in only a slight decrease in ROS, whereas higher concentrations led to a modest increase in ROS levels in HaCaT cells.

These results indicate that the *S. yangii* extract effectively inhibits UVB-induced ROS generation mainly in fibroblasts, with a concentration-dependent protective activity. This supports the potential use of the extract as an active ingredient in cosmetic formulations designed to protect skin cells from UV-induced oxidative stress. The divergent ROS responses observed in fibroblasts and keratinocytes likely reflect cell-type–specific antioxidant capacity and xenobiotic handling. Fibroblasts, which display lower basal antioxidant defenses than keratinocytes, exhibited a broader concentration window in which the extract effectively reduced UVB-induced ROS, consistent with a more favorable net redox balance [57,58,59]. In contrast, in HaCaT cells a significant effect was confined to 1.00%, suggesting tighter regulation of Nrf2/ARE signaling and/or faster phase-II metabolism of phenolic constituents [60]. The rise in ROS at ≥5.00% is consistent with the well-described biphasic behavior of polyphenols, where high concentrations promote extracellular H_2_O_2_ formation and redox cycling in the presence of trace metals [61,62]. Together, these data indicate a hormetic, concentration-dependent profile of the extract, with a wider therapeutic window in dermal fibroblasts than in keratinocytes.

The findings of this study suggest that the water–ethanol extract of *S. yangii* can effectively protect skin cells against oxidative stress triggered by UVB exposure. Several mechanisms may explain these observations. First, phenolic compounds abundant in the extract, particularly rosmarinic acid, are recognized for their capacity to neutralize reactive oxygen species (ROS), which are rapidly generated in skin cells exposed to UV. By directly scavenging these free radicals, the extract may limit cellular damage to essential macromolecules such as DNA, proteins, and lipids, which are otherwise susceptible to oxidative modification [18]. In addition to their direct antioxidant effects, compounds present in *S. yangii* may activate endogenous protective systems within cells. This could involve stimulating the expression or activity of key antioxidant enzymes—including superoxide dismutase, catalase, and glutathione peroxidase—which collectively contribute to maintaining redox balance under stress conditions [63,64]. Strengthening these intrinsic defense pathways enhances the skin’s resilience to oxidative injury. Another potential mechanism relates to the anti-inflammatory properties of polyphenolic constituents, which may interfere with signaling cascades that are typically activated by UVB, such as NF-κB and MAPK pathways. Suppressing these pathways can reduce the production of inflammatory mediators and matrix metalloproteinases (MMPs) that promote collagen breakdown and accelerate skin aging [65]. By modulating these cellular responses, the extract could help preserve the structural integrity of the skin and prevent long-term photodamage.

The obtained research results are an important addition to the available data in the context of the potential use of *S. yangii* extract, containing polyphenolic compounds, including a predominant amount of rosmarinic acid, in sunscreen preparations. Various studies have tested combinations of rosmarinic acid with well-known and widely used UV filters, including ethylhexyl methoxycinnamate (EHMC) [66,67,68], octocrylene (OCT), ethylhexyl salicylate (EHS) [66], avobenzone (BMDBM) [68], and bemotrizinol (BEMT) [67]. Rosmarinic acid has shown promising properties in improving photoprotection and mitigating oxidative stress. Research shows that combining natural antioxidant ingredients with well-known UVA/UVB sun filters can contribute to the development of innovative, safer, more effective, and more environmentally friendly sunscreen products with a reduced content of conventional UV filters in the final product [66,67,68].

### 2.6. Antimicrobial and Antibiofilm Activity of Extract

Skin infections are a common health problem that often requires long-term treatment with antibiotics. In addition, attention is paid to opportunistic pathogenic microorganisms, such as *Candida* spp., *Staphylococcus aureus*, or *Pseudomonas aeruginosa* [69,70]. *S. aureus* is considered the most common pathogen involved in skin infections worldwide, affecting patients of all ages across all geographic regions [71]. Similarly, *Streptococcus pyogenes* is recognized as one of the major bacterial causes of skin and soft tissue infections globally [72]. Antimicrobial resistance of pathogens is a global problem that has led to the development of research into natural materials as an alternative source of antibacterial and antibiofilm agents. Many plants, due to the presence of bioactive compounds, including flavonoids, phenolic acids, and tannins, exhibit strong antimicrobial properties, which makes them important materials for skin health. The antimicrobial effectiveness of plants is due to the synergy of bioactive compounds. Plant-derived substances are increasingly recognized as potential direct antimicrobials or modulators of bacterial resistance [73]. The search for new antimicrobial and antibiofilm agents is important not only for the prevention of skin diseases but also for their treatment as well [74].

This study represents the first comprehensive investigation into the antimicrobial properties and antibiofilm activity of water–ethanol herb extracts of *S. yangii* against Gram-positive bacteria, Gram-negative bacteria, and fungi. The extract exhibited antimicrobial activity in the range of 2–16 mg/mL (MIC). The highest sensitivity (MIC = 2 mg/mL) was observed for *Candida albicans*, *Bacillus subtilis*, and *Pseudomonas aeruginosa*, and the lowest for *Escherichia coli* ATCC 8739 (MIC = 16 mg/mL). It is also worth noting its effectiveness against *Staphylococcus aureus* and Streptococcus pyogenes, because while different types of skin infections may have different specific etiological factors, these two species of bacteria are particularly important. These findings are in line with previous reports on *Salvia* species: extracts typically show MIC values between 1.25 and 5 mg/mL against *S. aureus*, *Candida*, and *P. aeruginosa*, with Gram-positive bacteria generally more susceptible than Gram-negative [30]. The MBC/MFC values were generally higher than the MIC values, suggesting a bacteriostatic effect (Table 3).

The antibiofilm properties of *S. yangii* herb (more than 50% biofilm reduction) were only observed for three strains, namely, *Streptococcus agalactiae* PCM 2683, *Staphylococcus epidermidis* ATCC 8853, and *Staphylococcus aureus* 6538P, at concentrations of 4 mg/mL. Comparable results have been reported for other *Salvia* extracts: for example, *S. sclarea* extracts reduced *S. aureus* biofilms by near 50% [75]. In contrast, *P. aeruginosa* biofilms are generally more resistant to phytochemicals, which is consistent with our observation that no significant inhibition was recorded for this pathogen [76].

Our findings highlight the potential of *S. yangii* extract as an effective antimicrobial agent. Furthermore, the antibiofilm properties of the extract can be used to develop new strategies for the prevention and treatment of biofilm-related infections. This is in line with growing international interest in developing natural antibiofilm agents to address multidrug-resistant infections. Combination therapies of antibiotics with plant-derived compounds have also been shown to increase the effectiveness of biofilm eradication [77,78,79]. In cosmetics, extract of *S. yangii* can be used as a natural preservative or to allow the product to be described as antibacterial and/or antifungal. This effect may be related to the presence of polyphenolic compounds (rosmarinic acid, caffeic acid, and hesperidin) identified in the extract. The results of studies by other authors confirm that rosmarinic acid showed promising antibacterial and anticandidal properties, as well as antibiofilm activity. Its antifungal mechanisms included reduction in mitochondrial activity, a change in cell membrane integrity, and inhibition of protease production, while antibiofilm activity was linked to the reduction in exopolysaccharide production [80]. Other studies have shown that caffeic acid has a stronger antibacterial effect against staphylococcal strains than protocatechuic acid ethyl ester and catechin hydrate [81]. The molecular mechanism of the antibacterial action of caffeic acid is related to the interaction of this compound with the cell membrane, damage to its integrity and increased permeability, as well as disruption of the aerobic metabolism of *S. aureus* cells [82]. In turn, studies on hesperidin have shown that this flavonoid has antibiofilm activity against the synthesis of staphyloxanthin by methicillin-resistant *S. aureus* [83].

### 2.7. Rheology and Texture Analysis of Extract-Based Formulation

The first model form proposed in this study was a hydrogel. Hydrogels, which are based on a hydrophilic polymer in water, have a wide range of biomedical applications, and several polymer hydrogels can also be used in cosmetics, including for skin, hair, oral, and mucous membrane care. Hydrogels are created using gelling agents, which may be natural (e.g., agar, tragacanth, or alginate), semisynthetic (cellulose derivatives: methylcellulose or hydroxyethylcellulose), or synthetic (e.g., poly(acrylic acid) polymer (carbomer), poly(vinyl alcohol), or poly(ethylene glycol)), with the ability to swell in water and other suitable solvents [84,85,86]. Hydrogels have beneficial properties for cosmetic and dermatological applications: they are thixotropic, greaseless, easily spreadable, and non-comedogenic. In addition to their good organoleptic and aesthetic properties, an important advantage of hydrogels is the faster release of the active substance compared to creams and ointments. The use of hydrogels is recommended to accelerate skin regeneration and to support the treatment of skin diseases, including inflammation, acne vulgaris, fungal infections, or psoriasis, among other applications [85,87]. Carbopol^®^, used in the preparation of hydrogels, is a universal polymer widely used in cosmetic and pharmaceutical preparations. When in contact with water, they form hydrogel matrices with high viscosity under neutral pH conditions. They exhibit optimal textural, rheological, and bioadhesive properties [88]. Glycerol added to the formulation has an occlusive effect. It reduces transepidermal water loss and helps maintain the ordered liquid crystal phase of the lipid matrix [88,89].

A lotion was proposed as a second model cosmetic. Lotions are characterized by low viscosity and very high spreadability. This type of cosmetic should have a light consistency and spread pleasantly and easily, providing an even protective layer on the skin after application, without causing a feeling of greasiness or stickiness, and at the same time providing the impression of skin hydration. Additionally, moisturizing ingredients provide appropriate care. The formulation proposed in this study has a low content of fat phase ingredients, and a small amount of xanthan gum was also added to support the consistency of the cosmetic. The proposed lotion composition also provides appropriate skincare properties. Fatty ingredients: capric/caprylic triglycerides, ethylhexyl hydroxystearate, and isopropyl palmitate act as emollients and contribute to maintaining the skin’s proper level of lubrication. Furthermore, hydrophilic glycerin acts as a humectant and reduces transepidermal water loss in the skin. Owing to this composition, the lotion provides appropriate sensory properties during application while facilitating even distribution of active ingredients on the skin surface. In this case, the active ingredient is a *S. yangii* extract with antioxidant and UV radiation-absorbing properties, confirmed in our study.

**Figure 7:**
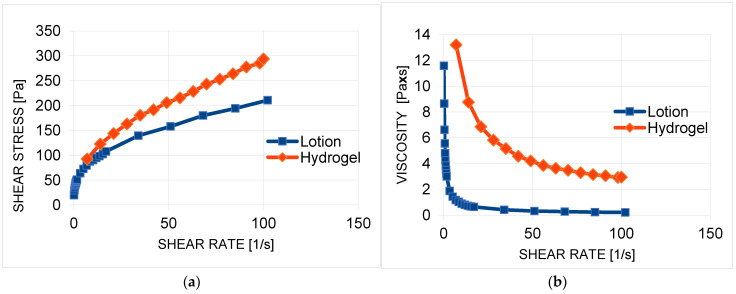
Flow curve (**a**) and viscosity curve (**b**) of formulations containing *S. yangii* extract.

Rheometric measurements were performed on the formulations containing the extract. A flow curve (Figure 7a) and a viscosity curve (Figure 7b) were determined at room temperature (25 °C). The curves showed that the preparations had a non-linear rheology, with a yield stress. In the range of shear rates tested, the apparent viscosity decreased (Table 4, Figure 7b), indicating that the formulations are non-Newtonian fluids diluted by shear.

Shear dilution in polymer formulations is caused by the ordering of the polymer chains under shear stress. At rest, the movement of polymer chains is chaotic and disordered, whereas under the influence of shear forces, they are oriented along the direction of flow. This parallel orientation of the chains reduces the internal friction resistance, thereby reducing the viscosity of the system [88]. A hydrogel containing *S. yangii* extract will spread very well on the skin and will not spill out when the container is inverted. A formulation with a yield stress of τ_0_ = 43.0 Pa will be adequately spreadable. The value obtained for this parameter is in line with other research results [88,90].

The results of the CRT (Compression/Relaxation/Tension) and ITPA (Instrumental Texture Profile Analysis) tests are shown in Table 5. The percentage of relaxation (%R) reflects the flexibility of the formulation—the higher the value, the lower the stiffness. The lotion showed a higher %R (92.60%) than the hydrogel. The lowest hardness 1 and hardness 2 values (easier application) were also observed for the lotion. Adhesiveness, important for the product’s retention in the skin and bioadhesiveness, was higher for the hydrogel. High cohesiveness values (>1) confirm the good structure and cohesion of both formulations. Formulations with high cohesiveness and adhesiveness adhere better to the skin. Higher elasticity was shown for the hydrogel (1.32), indicating its greater flexibility in regaining shape.

Texture parameters determine the sensory properties of the product. Hardness, cohesiveness, and elasticity influence the subjective impressions of people using the preparation. The results of the tests indicate that the formulations will spread easily on the skin, covering it evenly. The adhesion measurement results suggest that the preparations will remain at the application site, determining the bioavailability of the active substance. A correlation between rheological and textural parameters and product acceptance by users has been confirmed [91,92]. The results obtained indicate the high sensory quality of the hydrogel and lotion and their effective applicability.

## 3. Materials and Methods

### 3.1. Plant Material and Extraction

Experiments were carried out on the aerial parts of *S. yangii* harvested in August 2024, during the flowering stage, from the Botanical Garden (Kielce, Poland). A voucher specimen was deposited at the Herbarium KPC, Jan Kochanowski University, Kielce, Poland. The plant material was immediately subjected to drying in a convection oven (Binder FD 53, Tuttlingen, Germany) in an air stream at 35 °C and then ground in a mill (A11 basic, IKA-Werke, Staufen, Germany).

Ultrasound-assisted extraction (UAE) was performed using an extraction solvent (50/50 solution of water and ethanol (*v*/*v*), 60 mL), which was added to 2 g of powdered plant material. The extraction was conducted twice for 60 min using an ultrasonic bath (Polsonic 5, Warsaw, Poland). The resulting extracts were filtered using Whatman filter paper. Supercritical fluid extraction (SFE) was performed using a high-pressure micronization unit (SITEC-Sieber Engineering, Maur, Switzerland). The conditions for extraction from the material (100 g) were as follows: temperature 60 °C, pressure 40 MPa, and time 45 min. The carbon dioxide flow rate was maintained at a constant level of 10 kg/h. The extraction parameters were determined based on previous research [93,94,95].

### 3.2. Phytochemical Characteristics of Extract

The spectrophotometric methods (UV-1900i UV-Vis spectrophotometer, Shimadzu, Kyoto, Japan) were used to determine chemical components of *S. yangii* extracts obtained by ultrasound-assisted and supercritical fluid extraction.

The total polyphenol (TP) content was estimated by the Folin–Ciocalteu method [96]. Standard solutions of gallic acid with a precisely known concentration were used to construct a calibration graph. The analysis was performed in triplicate. The results were expressed as mg of gallic acid equivalents (GAEs) per mL of each extract. The absorbance was determined spectrophotometrically at 765 nm.

The total flavonoid (TF) content was estimated according to Kim et al. [97]. Standard solutions of catechin with a precisely known concentration were used to construct a calibration graph. The analysis was performed in triplicate. The results were expressed as mg of catechin equivalents (CEs) per mL of extract. The absorbance was determined at 510 nm.

The total phenolic acid (TPA) content was assessed according to Jain et al. [98]. Standard solutions of caffeic acid with a precisely known concentration were used to construct a calibration graph. The analysis was performed in triplicate. The results were expressed as mg of caffeic acid equivalents (CAEs) per mL of each extract. The absorbance was determined spectrophotometrically at 490 nm.

The condensed tannin (CT) content was estimated according to Tlili et al. [99]. Standard solutions of delphinidin with a precisely known concentration were used to construct a calibration graph. The analysis was carried out in triplicate. The results were expressed as mg of delphinidin equivalents (DpEs) per mL of each extract. The absorbance was determined at 550 nm.

### 3.3. HPLC Analysis of Extract

HPLC-DAD analysis was performed using a Hitachi Chromaster (Esprimo P240, Fujitsu, Tokyo, Japan) according to the procedure described by Dobros et al. [100]. The 40 mg of lyophilized extract was dissolved in 2 mL of methanol. Then, using an autosampler, 20 μL of extract was applied to a Purospher STAR RP-18e column (5 μm, 250 mm × 4.6 mm, Merck). The mobile phase contained 0.1% (*v*/*v*) formic acid dissolved in water (A) and 0.1% (*v*/*v*) formic acid dissolved in acetonitrile (Merck, Darmstadt, Germany) (B). The separation of compounds was carried out at 30 °C. A gradient of 10–20% B (0–35 min), 20–35% B (35–60 min), 35–10% B (60–60.1 min), and 10% B (60.1–70 min) was used at a flow rate of 1 mL/min. The analysis time was 70 min. HPLC analysis was performed in triplicate. The signal from a diode array detector was recorded in the form of a chromatogram at a wavelength of 310 nm.

Standard solutions of polyphenolic compounds were prepared by dissolving 1 mg of the standard in 1 mL of methanol, mixing, and filtering through a syringe filter (0.45 µm). HPLC analysis of the standard compounds (caffeic acid, hesperidin, and rosmarinic acid) was performed using the same procedure as for the *S. yangii* extract. The calibration curves, linear range, LOD, and LOQ for standard compounds were provided in Table S1 in the Supplementary Materials. The limit of detection (LOD) was calculated based on the equation: LOD = (3.3 × σ)/S, and the limit of quantification (LOQ): LOQ = (10 × σ)/S, where S is the slope of the standard curve and σ is the standard deviation of the point of intersection of the standard curve with the y-axis.

### 3.4. Antioxidant Activity Evaluation of Extract

The spectrophotometric methods (UV-1900i UV-Vis spectrophotometer, Shimadzu, Kyoto, Japan) were used to evaluate the antioxidant activity of *S. yangii* extract.

The method described by Benzie and Strain [101] was used to determine the Ferric-Reducing Antioxidant Power (FRAP). The absorbance was measured at a wavelength of 593 nm. The analysis was conducted on three separate occasions for each sample, and the results were expressed in mmol of Trolox equivalent (TE) per L of extract. A calibration curve was constructed by analyzing the samples at six concentrations, ranging from 0.031 to 1 mmol TE/L.

The anti-radical power of the extract was measured by the widely used 2,2-diphenyl-1-picrylhydrazyl test [102]. The absorbance was measured at a wavelength of 517 nm. Each sample was analyzed on three separate occasions. The following formula was used to calculate the degree of inhibition of DPPH radical by the sample: DPPH scavenging activity (%) = [A_0_ − A_1_/A_0_] × 100%, where A_0_ is the absorbance of the control and A_1_ is the absorbance of the sample. In both antioxidant tests, a solution of rosmarinic acid in ethanol (1 mg/mL), diluted 40-fold (25 μg/mL), was used as a positive control.

### 3.5. Cell Culture

The HaCaT keratinocyte and HDF fibroblast human cell lines, sourced from CLS Cell Lines Service (Eppelheim, Germany), were routinely cultured under standard in vitro conditions. Cells were grown in Dulbecco’s Modified Eagle Medium (DMEM; Biological Industries, Cromwell, CO, USA), which included phenol red, L-glutamine, sodium pyruvate, and glucose at a final concentration of 4.5 g/L. To support optimal growth and viability, the medium was enriched with 10% fetal bovine serum (FBS) and an antibiotic mix consisting of penicillin (100 U/mL) and streptomycin (1000 µg/mL), both provided by Thermo Fisher Scientific (Waltham, MA, USA). The cultures were incubated at 37 °C in a humidified environment with 5% CO_2_. For experimental assays measuring oxidative stress (ROS generation), mitochondrial metabolic activity (resazurin assay), and lysosomal membrane integrity (neutral red uptake assay), cells were plated into 96-well microplates at a density of 10,000 cells per well. After a 24 h preincubation period to allow for cell adhesion and equilibration, the cells were exposed to varying concentrations of the plant extract, which was freshly prepared in DMEM.

### 3.6. Assessment of Cytotoxicity—Alamar Blue (AB) and Neutral Red (NR) Uptake Assays

The cytotoxic potential of *S. yangii* extract was evaluated using two complementary in vitro assays: the Alamar Blue test, based on the metabolic conversion of resazurin sodium salt (Merck KGaA, Darmstadt, Germany), and the Neutral Red assay, which assesses lysosomal dye accumulation in viable cells (Merck KGaA, Darmstadt, Germany), following the protocol described previously [103]. For both assays, human keratinocytes and fibroblasts were seeded into sterile 96-well flat-bottom plates. Alamar Blue measurements were performed using black microplates, while Neutral Red assays were conducted in transparent plates (Googlab Scientific, Rokocin, Poland). After a 24 h cell attachment phase, cultures were exposed to serial dilutions of the tested extract (ranging from 0.01% to 10.00%), prepared freshly in DMEM medium. Control groups consisted of untreated cells maintained in DMEM alone. In the case of the Alamar Blue test, after 24 h of treatment, the culture medium containing the extract was removed and replaced with 60 μM resazurin solution. Cells were incubated for 2 h at 37 °C, after which fluorescence was measured at 570 nm using a microplate reader (Thermo Fisher Scientific, Waltham, MA, USA). For the Neutral Red assay, the extract-containing medium was discarded and substituted with a 40 μg/mL solution of the NR dye. After a 2 h incubation at 37 °C, the cells were washed twice with phosphate-buffered saline (PBS; Genos, Łódź, Poland), followed by the addition of a destaining solution composed of ethanol, acetic acid, and water (50%/1%/49%). The plates were shaken at room temperature for 15 min before spectrophotometric readings were taken at 540 nm. Viability of untreated cells (HaCaT and HDF) was considered as 100%, and all extract concentrations were analyzed in three separate experiments, each performed in triplicate.

### 3.7. Evaluation of Intracellular Reactive Oxygen Species (ROS) Levels in Skin Cells Without and After UVB Radiation Exposure

To investigate the potential of the *S. yangii* extract to mitigate intracellular reactive oxygen species (ROS) generation in cultured human skin cells, the fluorogenic indicator 2′,7′-dichlorodihydrofluorescein diacetate (H_2_DCFDA; Thermo Fisher Scientific, Waltham, MA, USA) was employed, following the method described previously [95]. Initially, HaCaT keratinocytes and HDF fibroblasts were plated separately into black 96-well microplates and allowed to adhere for 24 h under standard culture conditions. Subsequently, the medium was exchanged for DMEM (VWR International, Radnor, PA, USA) containing various concentrations of the tested extract (ranging from 0.01% to 10.0%), and the cells were incubated for another 24 h. After this treatment period, the culture media were carefully aspirated, and the cells were rinsed twice with sterile phosphate-buffered saline (PBS). Cells were then exposed to UVB irradiation at a dose of 0.5 J/cm^2^ using a UV crosslinker device (UVP CL-3000M; Analytik Jena, Jena, Germany). Cells plated on a separate plate were not irradiated and served as negative controls. Following UVB exposure, the PBS was removed from all wells, and cells from both irradiated and control plates were incubated with 200 μL of a 10 μM solution of H_2_DCFDA prepared in serum-free DMEM. The fluorescence of the oxidized form of the probe (DCF) was detected using a microplate reader (FilterMax F5, Thermo Fisher Scientific, Waltham, MA, USA), with excitation and emission wavelengths set at 485 nm and 535 nm, respectively.

### 3.8. Determination of Anti-Collagenase and Anti-Elastase Activity of Extract

To evaluate the anti-aging potential of *S. yangii* extract, their influence on the intracellular levels of collagenase and elastase enzymes was examined in HDF fibroblasts treated with extract concentrations of 0.1% and 1.0%. Cells were seeded into 6-well culture plates at a density of 1 × 10^4^ cells per well and incubated with the specified extract dilutions for a period of 24 h. Following treatment, the cells were rinsed twice with sterile phosphate-buffered saline (PBS), after which 150 μL of RIPA lysis buffer was added to each well to obtain cellular lysates. These lysates were then subjected to quantitative analysis of collagenase and elastase content using commercially available ELISA kits: Human COL2α1 (collagenase) and Human Ne/ELA2 (elastase), both obtained from Elabscience Biotechnology Inc. (Houston, TX, USA). All steps were performed by the protocols provided by the manufacturer.

To validate the enzymatic inhibition, known reference inhibitors were employed: succinyl–alanyl–alanyl–prolyl–valine–chloromethyl ketone (SPCK; Adooq Bioscience, Irvine, CA, USA) at a final concentration of 30 µM served as the positive control for elastase inhibition, while 1,10-phenanthroline (Abcam, Cambridge, UK) at a final concentration of 300 µM was used for collagenase inhibition. The standard curves for the reference standards of collagenase and elastase were prepared according to the manufacturer’s instructions, within the concentration ranges of 0 to 40 ng/mL and 0 to 50 ng/mL, respectively. The absorbance of each well was recorded at 450 nm using a microplate reader (FilterMax F5, Thermo Fisher Scientific, Waltham, MA, USA). All measurements were performed in triplicate for each tested concentration of the extract.

### 3.9. Determination of Anti-Tyrosinase Activity of Extract

The anti-tyrosinase potential of the *S. yangii* extract, as an indicator of its ability to suppress melanin biosynthesis, was examined following the method outlined by Krochmal-Marczak et al. [104]. Briefly, 20 μL of the plant extract, prepared at concentrations of 0.1% and 1.0%, were pipetted into individual wells of a 96-well microplate. Subsequently, 500 U/mL of mushroom-derived tyrosinase (Sigma–Aldrich, Poznań, Poland) was added, followed by 120 μL of phosphate buffer solution (100 mM, pH 6.8). The mixture was gently shaken and incubated in the dark for 10 min at ambient temperature (22–24 °C). Next, 40 μL of L-DOPA solution (4 mM; Merck KGaA, Darmstadt, Germany) was introduced into each well to initiate the enzymatic reaction. As a reference control representing 100% tyrosinase activity, distilled water was used instead of the plant extract, in combination with the enzyme, substrate, and buffer. All reaction mixtures were then incubated under light-protected conditions for an additional 20 min. The formation of dopachrome, an indicator of tyrosinase activity, was monitored spectrophotometrically at 475 nm using a microplate reader (FilterMax F5; Molecular Devices, San Jose, CA, USA). Kojic acid at a concentration of 500 μg/mL served as a positive control inhibitor of tyrosinase. Absorbance values were corrected by subtracting the background absorbance of samples lacking both tyrosinase and substrate. Each tested sample was analyzed in three replicates. The percentage inhibition of tyrosinase activity was calculated using the following equation:

$$\%\;\mathrm{Tyrosinase}\;\mathrm{inhibition}=\frac{\mathrm{Abs}\_\mathrm{control}-\mathrm{Abs}\_\mathrm{sample}}{\mathrm{Ab}\_\mathrm{control}}\;\times \;100\%$$

### 3.10. Evaluation of the Antimicrobial Activity and Biofilm Formation Inhibition of Extract

The antimicrobial properties of the extract were evaluated against 15 microorganisms, comprising Gram-positive and Gram-negative bacterial strains and one fungal strain, all obtained from the Polish Collection of Microorganisms (Wrocław, Poland). The minimum inhibitory concentration (MIC) was determined using a broth microdilution assay, according to the recommendations of the Clinical and Laboratory Standards Institute (CLSI) [105]. Overnight bacterial and fungal cultures were diluted 1:10 in fresh Mueller–Hinton broth. Initially, 100 µL of the extract (33 mg/mL) was added to the first well of a sterile 96-well microplate, followed by serial two-fold dilutions with inoculated broth to achieve final concentrations of 0.25–16 mg/mL. Plates were incubated under aerobic conditions at 35 °C for 18–20 h. After incubation, resazurin (0.1 µg/mL) was added to each well to assess viability. A blue color indicated growth inhibition, whereas a pink color indicated viable cells, based on a method adapted from Sarker et al. [106].

The minimum bactericidal and fungicidal concentration (MBC/MFC) was determined by plating 100 µL aliquots from wells showing no visible growth in the MIC assay onto solid BHI medium. Following incubation at 35 °C for 18–20 h, the MBC/MFC was defined as the lowest extract concentration that eliminated ≥99.9% of the initial microbial inoculum.

The extract’s ability to inhibit biofilm formation was assessed using a modified protocol from Merritt et al. [107]. Microbial suspensions were incubated in 96-well microplates with 100 µL of Mueller–Hinton broth supplemented with the extract at concentrations ranging from 0.25 to 16 mg/mL. After incubation at 37 °C for 24 h, non-adherent cells were discarded, and the wells were washed with sterile distilled water. Adhered biofilms were stained with 0.1% crystal violet for 15 min. Excess stain was removed by washing, and the bound dye was solubilized in 95% ethanol. Absorbance was measured at 595 nm using an Infinite M200 PRO microplate reader (Tecan, Männedorf, Switzerland). All experiments were performed in triplicate. Biofilm inhibition was calculated as a percentage using the following formula [108]:

$$\%\;\mathrm{Biofilm}\;\mathrm{inhibition}=\frac{\mathrm{control}\;\mathrm{OD}\;\mathrm{value}-\mathrm{treated}\;\mathrm{OD}\;\mathrm{value}}{\mathrm{control}\;\mathrm{OD}\;\mathrm{value}}\;\times \;100\%$$

### 3.11. Determination of Model Cosmetics Containing Extract

#### 3.11.1. Preparation of Formulations

**Figure 8:**
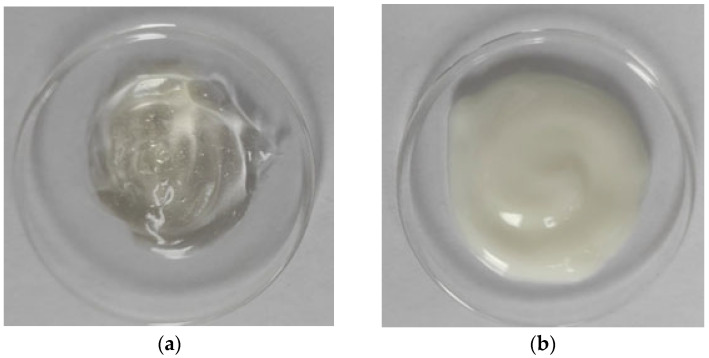
Formulations containing *S. yangii* extract: hydrogel (**a**) and emulsion (**b**).

The hydrogel was prepared based on Carbopol^®^ UltrezTM 10 (Lubrizol, Cleveland, USA). Carbomer (0.5%) was dispersed in a mixture of water and glycerol (3.0%) and stirred until completely dissolved. The formulation was then neutralized with triethanolamine to a pH = 7.0, resulting in a transparent hydrogel. Then, *S. yangii* extract (5.0%) was introduced into the hydrogel mechanically (Figure 8).

The lotion was prepared using the classic hot emulsification method, which involves heating and mixing the components of both phases separately. The oil phase (%*w*/*w*) consisted of capric/caprylic triglycerides (Croda Inc., Yorkshire, United Kingdom) (7.0), cetearyl alcohol (SABO S.p.A, Rome, Italy) (3.0), ceteareth-20 (BASF Personal Care) (4.0), polysorbate-20 (Croda Inc., Yorkshire, United Kingdom) (1.0), ethylhexyl hydroxystearate (Croda Inc., Yorkshire, United Kingdom) (1.0), and isopropyl palmitate (Croda Inc., Yorkshire, United Kingdom) (1.0), while the aqueous phase (%*w*/*w*) consisted of glycerine (Brenntag) (3.0), preservatives: sodium benzoate and potassium sorbate (Akema Fine Chemicals, Rome, Italy) (0.5), xanthan gum (Sigma-Aldrich, Germany) (0.3), and distilled water (up to 100). The two phases were combined at 70 °C, and then the entire system was mixed and cooled simultaneously. The *S. yangii* extract (5.0 %*w*/*w*) was added after lowering the temperature to 40 °C (Figure 8). Samples were stored for 1 month after preparation. During this time, their stability was analyzed using a Turbiscan stability analyzer (TURBISCAN Lab Expert, Formulation Company, Paris, France). The operating principle and methodology have been described in detail in a previous study [109]. No signs of system instability were observed during the entire storage period.

The pH was monitored using a SevenCompact^TM^ S210 laboratory pH meter equipped with an InLaB^®^Expert Pro-ISM electrode (Mettler-Toledo International Inc., Greifensee, Switzerland).

#### 3.11.2. Rheological Properties of Extract-Based Formulation

To assess the rheological properties of the hydrogel, the rotational rheometry method was applied using a Lamy RM 200 Touch rheometer (Lamy Rheology Instruments, Champagne au Mont d’Or, France), equipped with an MK-CP 2445 measuring system and a CP-1 Plus thermostat. Measurements were carried out in a plate-to-plate arrangement at 25 ± 1 °C. Flow and viscosity curves were recorded in controlled shear rate mode, from 5.0 to 100.0 s^−1^ for 15 min.

The dependence of shear stress and viscosity on the shear rate of the lotion was determined using a Brookfield HADV-III Ultra rheometer (Brookfield Engineering Laboratories, INC., Middleboro, MA, USA), in a plate-cone system. The tests were carried out using RheoCalc software (version 3.2), which performs automatic measurements at given shear rates and records the results. Measurements were carried out at 25 °C.

#### 3.11.3. Textural Properties of Extract-Based Formulation

Hydrogel and lotion samples were analyzed using a TX-700 texture analyzer (Lamy Rheology, France) equipped with an 8 mm diameter hemispherical probe. Measurements were performed in the Compression/Relaxation/Tension (CRT) and Instrumental Texture Profile Analysis (ITPA) modes. CRT analysis was performed with the following parameters: down speed 0.5 mm/s, force to start 0.05 N, relaxation time 20 s, and wait position 20 mm. The ITPA cycle was performed with the following parameters: down speed 1 mm/s, force to start 0.05 N, distance 5 mm, and wait position 10 mm. Measurements were taken at a temperature of 25 ± 0.1 °C. RheoTex software dedicated to the TX-700 analyzer (version TX-UK01/2019) was used to record and analyze the results. The theoretical basis for texture analysis has been presented in detail in a previous publication [88].

### 3.12. Statistical Analysis

Statistical analysis for phytochemical composition and antioxidant activity of *S. yangii* extract was performed using TIBCO Statistica 13.3 (StatSoft, Krakow, Polska). Identification of means with significant differences (*p* < 0.05) was performed using one-way analysis of variance (ANOVA).

Rheological and textural data analysis was performed using the two-sided Student’s *t*-test in Statistica 12.0 (Statsoft, Krakow, Poland). Results with a *p* < 0.05 were considered statistically significant. Results that did not meet this criterion were denoted by the abbreviation NS. Results are given as mean values with standard deviation (SD).

## 4. Conclusions

In the present study, we examined extracts from a little-known and insufficiently researched plant from the Lamiceae family. Extracts of *Salvia yangii* obtained by ultrasound-assisted extraction had higher content of polyphenolic compounds compared to those obtained by supercritical CO_2_ extraction. Two phenolic acids (rosmarinic and caffeic acids) and one flavonoid (hesperidin) were confirmed in the water–ethanol *S. yangii* herb extract. The results of in vitro studies indicate that the extract exerts a non-toxic and even stimulating effect on skin cells (both fibroblasts and keratinocytes) and suggest that the use of the extract in appropriately low concentrations (0.01% and 0.10%) may be beneficial for skin cell viability. UAE exhibited bacteriostatic and anti-biofouling properties. In cosmetics, this extract can be used as a natural preservative or to allow the product to be described as antimicrobial. A significant scope of research on the *S. yangi* extract, indicating its multifaceted applications, is concerned with its anti-aging properties. Preventing and combating the signs of skin aging (e.g., loss of firmness and elasticity, wrinkles, and hyperpigmentation) is an important direction of modern research on active ingredients of cosmetics. Key in this respect are studies on the ability to inhibit enzymes such as collagenase (can degrade collagen, the fibrous component of the extracellular matrix (ECM), and the major structural protein in human skin), elastase (enzyme involved in the degradation of elastin, a protein responsible for skin elasticity), and tyrosinase (enzyme in the biosynthesis of melanin, responsible for catalysis of the first two synthesis reactions, i.e., hydroxylation of tyrosine to DOPA and oxidation of DOPA to dopaquinone). *S. yangii* extract was shown to be a promising active material for the cosmetic industry due to its anti-collagenase, anti-elastase, and anti-tyrosinase activity. Increasing the concentration of the *S. yangii* extract leads to a stronger inhibition of these enzymes. The extract can also be used as an active ingredient in cosmetic formulations, protecting the skin against ROS, oxidative stress, and UVB radiation. The study results indicate that the extract exhibits photoprotective and ROS-scavenging effects at lower concentrations (from 0.01% to 2.5%), effectively protecting fibroblasts and keratinocytes from UVB-induced oxidative damage. It can be concluded that *S. yangii* can be used in cosmetics, e.g., for mature skin with signs of aging such as wrinkles or age spots. A wide range of research in this area is presented in this publication. However, further scientific research is needed to confirm the potential applications of cosmetic formulations based on *S. yangii* extract, e.g., instrumental in vivo evaluation of various skin parameters, including cutometer, indentometer, tewameter, or skin visiometer for anti-aging effects and mexameter or skin-colorimeter for photoprotection properties and depigmenting effects. Given the growing expectations of consumers regarding the application values of cosmetic products, sensory and organoleptic assessment (including, among others, color, smell, consistency, viscosity, stickiness, and spreadability) of the tested formulations would also be important.

## Figures and Tables

**Table 1:** Phytochemical composition of *S. yangii* extracts (mg/mL ± SD).

Extract	TP	TF	TPA	CT
UAE	64.46 ± 0.09 ^a^	17.39 ± 0.06 ^a^	4.92 ± 0.04 ^a^	4.12 ± 0.03 ^a^
SFE	4.29 ± 0.25 ^b^	1.43 ± 0.08 ^b^	0.96 ± 0.02 ^a^	0.74 ± 0.02 ^a^

UAE, ultrasound-assisted extraction; SFE, supercritical fluid extraction; TP, total polyphenols expressed as gallic acid equivalents (GAEs); TF, total flavonoids expressed as catechin equivalents (CEs); TPA, total phenolic acids expressed as caffeic acid equivalents (CAEs); CT, condensed tannins expressed as delphinidin equivalents (DpEs); SD, standard deviation. The letters a and b indicate statistically significant differences at *p* < 0.05 according to ANOVA.

**Table 2:** Content of polyphenolic compounds present in *S. yangii* water–ethanol extract (UAE).

Number	Compound	Retention Time [min]	[mg/mL] ± SD
1	Caffeic acid	17.8	0.04 ± 0.00
2	Hesperidin	43.9	0.16 ± 0.01
3	Rosmarinic acid	45.9	1.32 ± 0.04

**Table 3:** MIC and MBC/MFC values of *S. yangii* extract in mg/mL.

Test Microorganism	MIC	MBC/MFC
*Candida albicans* ATCC10231	2	16
*Streptococcus agalactiae* PCM 2683	8	8
*Enterococcus faecalis* PCM 2784	8	8
*Proteus mirabilis* ATCC 29906	8	16
*Streptococcus mutans* ATCC 25175	4	8
*Staphylococcus epidermidis* ATCC 8853	8	8
*Streptococcus pyogenes* ATCC 19615	8	16
*Escherichia coli* UPEC PCM 176	8	8
*Enterococcus hirae* ATCC 10541	8	16
*Bacillus subtilis PCM 486*	2	16
*Staphylococcus aureus 6538P*	4	4
*Staphylococcus epidermidis* PCM 2118	4	4
*Escherichia coli* ATCC 8739	16	16
*Pseudomonas aeruginosa* PAO1	2	8
*Ralstonia solanacearum* Z1	4	4

MIC, minimum inhibitory concentration; MBC, minimum bactericidal concentration; MFC, minimum fungicidal concentration; UPEC, uropathogenic *E. coli.*

**Table 4:** Viscosity values at different shear rates (mean ± SD, *n* = 10, T = 25 °C ± 0.1 °C).

Shear Rate [s^−1^]	Hydrogel Viscosity [Paxs]	Lotion Viscosity [Paxs]	*p*
30	5.16 ± 0.09	0.49 ± 0.06	*p* < 0.05
50	3.86 ± 0.10	0.30 ± 0.05	*p* < 0.05
100	2.55 ± 0.01	0.21 ± 0.01	*p* < 0.05

**Table 5:** Texture parameters of hydrogel and lotion containing *S. yangii* extract (mean ± SD, *n* = 3, T = 25 °C ± 0.1 °C).

Parameter	Hydrogel	Lotion	*p*
Relaxation [%]	79.90 ± 0.87	92.60 ± 13.15	NS
Hardness 1 [N]	0.11 ± 0.02	0.05 ± 0.00	*p* < 0.05
Hardness 2 [N]	0.13 ± 0.01	0.05 ± 0.00	*p* < 0.05
Cohesiveness [-]	1.45 ± 0.19	1.31 ± 0.06	NS
Adhesiveness [mJ]	0.40 ± 0.10	0.20 ± 0.00	NS
Elasticity [-]	1.32 ± 0.12	0.85 ± 0.00	*p* < 0.05

## Data Availability

The original contributions presented in this study are included in the article.

## Supplementary Materials

The following supporting information can be downloaded at: https://www.mdpi.com/article/10.3390/molecules30173505/s1, Figure S1: UV–Vis spectra of the reference substances; Figure S2: HPLC chromatogram at 330 nm of the reference substances mixture. Numbering of polyphenolic compounds: 1-caffeic acid, 2-hesperidin, 3-rosmarinic acid; Table S1: The calibration curves, linear range, LOD, and LOQ for the compound present in *Salvia yangii* water–ethanol extract.

## Abbreviations

The following abbreviations are used in this manuscript:

UAE	Ultrasound-assisted extraction
SFE	Supercritical fluid extraction
TP	Total polyphenol
GAE	Gallic acid equivalents
TF	Total flavonoid
CE	Catechin equivalents
TPA	Total phenolic acid
CAE	Caffeic acid equivalents
CT	Condensed tannin
DpE	Delphinidin equivalents
EHMC	Ethylhexyl methoxycinnamate
OCT	Octocrylene
EHS	Ethylhexyl salicylate
BMDBM	Avobenzone
BEMT	Bemotrizinol
FRAP	Ferric Reducing Antioxidant Power
TE	Trolox equivalent
DPPH	2,2-diphenyl-1-picrylhydrazyl
TPTZ	2,4,6-tripyridyl-s-triazine
DMEM	Dulbecco’s Modified Eagle’s Medium
MIC	Minimal Inhibitory Concentration
CLSI	Clinical and Laboratory Standards Institute
MBC	Minimum Bactericidal Concentration
MFC	Minimum Fungicidal Concentration
AB	Alamar Blue
NR	Neutral Red
ROS	Reactive Oxygen Species
CRT	Compression/Relaxation/Tension
ITPA	Instrumental Texture Profile Analysis
SPF	Sun Protection Factor
CF	Correction factor
EE	Erythemogenic effect
