# Supplementary material for: Evaluation of Salvia yangii Extract as a Promising Protective Raw Material Applied Topically to the Skin
Source: Molecules. 2025 Aug 27;30(17):3505. doi: 10.3390/molecules30173505 (PMC12430148; doi:10.3390/molecules30173505)
Supplement: Supplementary file 1 [file molecules-30-03505-s001.zip › molecules-3760903-supplementary.pdf]

Table S1. The calibration curves, linear range, LOD, and LOQ for the compound present in *Salvia yangii* water–ethanol extract.

Number	Compound	Calibration curve	R^2	Linear range [mg/mL]	LOD [mg/mL]	LOQ [mg/mL]
1	Caffeic acid	y = 49472514.87x + 2055.42	0.999	0.01 – 0.12	0.01	0.02
2	Hesperidin	y = 15079884.64x +58174.48	0.999	0.01 – 0.08	0.08	0.23
3	Rosmarinic acid	y = 28614208.85x – 9260.92	0.998	0.01 – 0.40	0.03	0.08

y – area under the curve [AU], x – compound concentration [mg/mL]

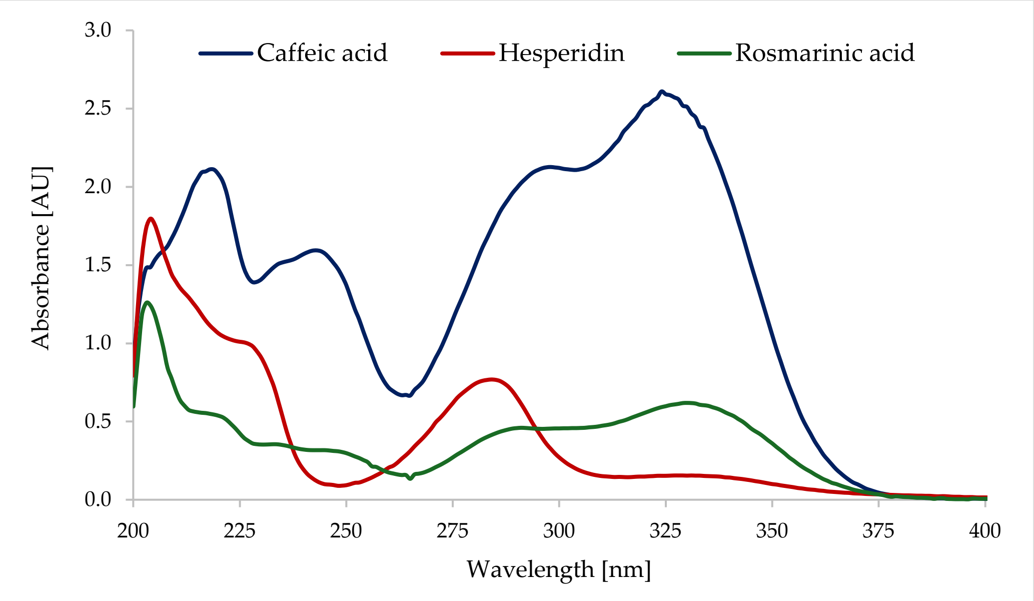

Figure S1. UV–Vis spectra of the reference substances.

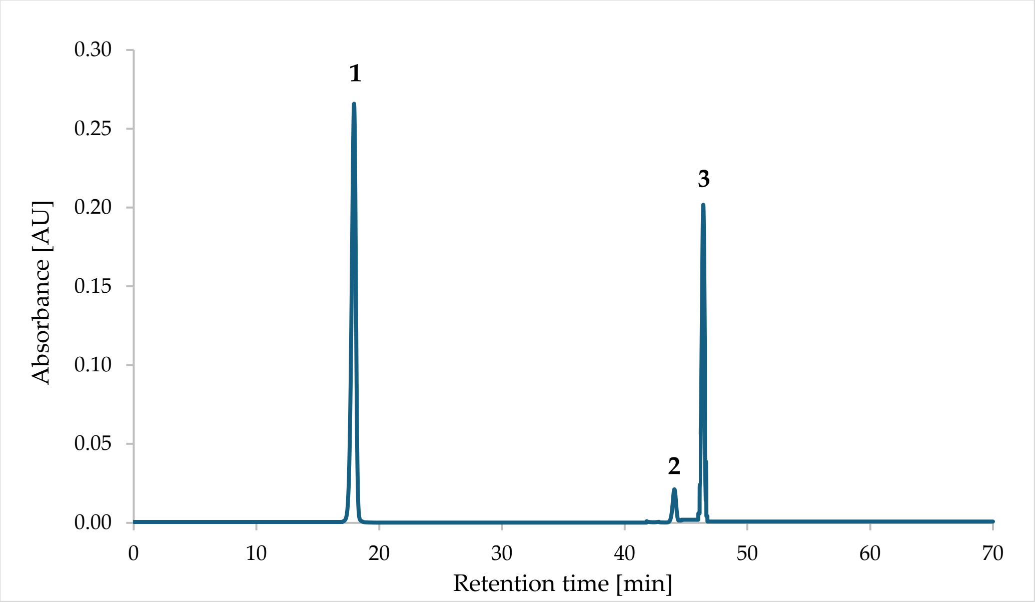

Figure S2. HPLC chromatogram at 330 nm of the reference substances mixture. Numbering of polyphenolic compounds: 1-caffeic acid, 2-hesperidin, 3-rosmarinic acid.
